# Effective coverage and budget implications of skill-mix change to improve neonatal nursing care: an explorative simulation study in Kenya

**DOI:** 10.1136/bmjgh-2019-001817

**Published:** 2019-12-02

**Authors:** Apostolos Tsiachristas, David Gathara, Jalemba Aluvaala, Timothy Chege, Edwine Barasa, Mike English

**Affiliations:** 1 Nuffield Department of Population Health, University of Oxford, Oxford, UK; 2 Health Services Unit, KEMRI - Wellcome Trust Research Programme, Nairobi, Kenya; 3 Department of Paediatrics and Child Health, University of Nairobi, Nairobi, Kenya; 4 Institute of Healthcare management, Strathmore University, Nairobi, Kenya; 5 Health Economics Research Unit, Centre for Geographic Medicine Research Coast, Nairobi, Kenya; 6 Nuffield Department of Medicine, Centre for Tropical Medicine and Global Health, University of Oxford, Oxford, UK

**Keywords:** child health, health economics, health policy, health services research, public health

## Abstract

**Introduction:**

Neonatal mortality is an urgent policy priority to improve global population health and reduce health inequality. As health systems in Kenya and elsewhere seek to tackle increased neonatal mortality by improving the quality of care, one option is to train and employ neonatal healthcare assistants (NHCAs) to support professional nurses by taking up low-skill tasks.

**Methods:**

Monte-Carlo simulation was performed to estimate the potential impact of introducing NHCAs in neonatal nursing care in four public hospitals in Nairobi on effectively treated newborns and staff costs over a period of 10 years. The simulation was informed by data from 3 workshops with >10 stakeholders each, hospital records and scientific literature. Two univariate sensitivity analyses were performed to further address uncertainty.

**Results:**

Stakeholders perceived that 49% of a nurse full-time equivalent could be safely delegated to NHCAs in standard care, 31% in intermediate care and 20% in intensive care. A skill-mix with nurses and NHCAs would require ~2.6 billionKenyan Shillings (KES) (US$26 million) to provide quality care to 58% of all newborns in need (ie, current level of coverage in Nairobi) over a period of 10 years. This skill-mix configuration would require ~6 billion KES (US$61 million) to provide quality of care to almost all newborns in need over 10 years.

**Conclusion:**

Changing skill-mix in hospital care by introducing NHCAs may be an affordable way to reduce neonatal mortality in low/middle-income countries. This option should be considered in ongoing policy discussions and supported by further evidence.

**Key questions**

**What is already known?**

- The neonatal mortality target in United Nations’ Sustainable Development Goals is far from being achieved in many low/middle-income countries.

**What are the new findings?**

- If current rates of expenditure on neonatal nursing continue into the future, >95% of sick newborns requiring hospital care will receive poor quality care that is likely to minimise improvements in neonatal mortality and prevent any benefits accruing from investments in additional technological solutions.
- A main driver for improving quality of care in neonatal care is the employment of substantially more nurses.
- A combination of nurses and neonatal healthcare assistants is likely to be the most affordable option to improve the quality of neonatal care within any given budget for nursing staff.
- The neonatal nursing budget should be 14 times larger to provide effective coverage to all newborns in need over 10 years compared with the projected current budget level.

**What do the new findings imply?**

- Ongoing policy discussions should consider changing the current skill-mix in neonatal care by employing more nurses and introducing healthcare assistants.

## Introduction

Worldwide, newborns account for the largest proportion of deaths under the age of 5. Almost 99% of the 2.5 million neonatal deaths occur in low/middle-income countries (LMICs).^1^ As a result, tackling neonatal mortality is an urgent policy priority to improve global population health and reduce health inequality.^2^ However, the United Nations’ Sustainable Development Goals that include a specific neonatal mortality target is far from being achieved in many countries in sub-Saharan Africa.^3^

Several facility-based quality improvement initiatives have been implemented to reduce neonatal mortality in LMICs.^4^ Little attention has, however, been paid to workforce innovations or interventions, although nurse-to-patient ratios are strongly related to neonatal mortality.^5^ It is likely that high neonatal mortality in sub-Saharan Africa may be partially attributed to lack of access to appropriate neonatal nursing care with evidence from Kenya of inadequate nursing numbers in the public sector,^6^ deficits in nursing knowledge in neonatal hospital units,^7^ and limited access to overburdened neonatal hospital care (only 25% of newborns in need in Nairobi access a hospital ready to provide high-quality care).^8 9^

While efforts must be made to tackle these problems and expand the professional nursing workforce providing neonatal care, evidence from high-income and LMICs in other arenas shows that innovations in professional roles and task delegation in healthcare may save costs^10^ and support expansion in delivery of quality care.^11 12^ As health systems in Kenya and elsewhere seek to tackle increased neonatal mortality by improving the quality of care one option is to train and employ neonatal healthcare assistants (NHCAs) to support professional nurses by taking up low-skill tasks. This could free-up time for nurses to focus on more complex tasks. As part a larger set of work conducted in collaboration with the Nairobi City County,^13 14^ which has almost double the national average neonatal mortality rate (39 vs 22 per 1000 live births),^15^ we aimed to explore how different hypothetical models of skill-mix to provide neonatal nursing care would impact on care quality and budgets.

## Methods

### Setting

Our analysis focused on Nairobi City County with a population of ~4.5 million people, 60%–70% of whom live in slums or low-income areas.^16 17^ Although the proportion of births in health facilities is well above the national average (89% vs 61%), neonatal mortality in Nairobi is much higher than elsewhere in Kenya.^18^ In prior work, we have demonstrated that four public hospitals currently provide care to 71% of all sick newborns being admitted to facilities capable of providing care 24 hours a day 7 days a week in Nairobi City County.^19^ The national and county government finance neonatal care in these hospitals as part of a national free maternity care programme, although in one tertiary hospital copayments are required from families.^20 21^

### Alternative strategies for addressing workforce deficits

This study estimates future nursing staff costs of different skill-mix alternatives to achieve better quality of care and higher coverage without incorporating any other broader costs. In prior work, we obtained data on the existing nursing workforce and nurse to patient (baby) ratios in the four public hospitals.^19^ These data provided the starting point for considering five alternative courses of action (hereafter called models) that illustrate for Kenyan health policymakers the possible impact of introducing NHCAs on budgets and effective coverage.

Model 1 was regarded as a base case, where neonatal care continued to be delivered at current staffing and quality levels (ie, usual care). Model 2 and model 3 explored different skill-mix solutions to expand the workforce to achieve acceptable staff-to-newborn ratios (table 1) in existing health facilities but without any expansion of the ability of the health system to close the coverage gap identified in earlier work. In model 2, acceptable staff-to-patient ratios are achieved only by increasing the number of neonatal nurses while in model 3 they are achieved through a combination of neonatal nurses and NHCAs. In additional work, model 4 assumes that a combination of neonatal nurses and NHCAs is used to expand coverage so that the public sector workforce is sufficient to provide care for 71% of all newborns in need of inpatient hospital care in Nairobi (based on the existing public sector contribution to service delivery and assuming 29% of care will be in the private or not-for-profit sector), while model 5 assumes a suboptimal approach (half-way) to improvement of staff-to-newborn ratios with current coverage levels based on model 3. An overview of the five alternative models with regard to their expected level of quality of care, coverage of newborns in need and inclusion of NHCAs is presented in table 1.

**Table 1:** Description of the alternative models

Alternative strategies	Description	Skill-mix	Newborn-to-staff ratio	Percentage of newborns receiving good quality nursing care	Coverage level of all newborns in need for hospital care
Model 1	Care as usual, where neonatal care continued to be delivered at current staffing and quality levels.	Only neonatal nurses	15 in all types of care	0%	39%
Model 2	This model achieves good quality of nursing care at current levels of coverage by increasing the number of neonatal nurses.	Only neonatal nurses	6 in standard care 3 in intermediate care 1 in intensive care	100%	39%
Model 3	This model achieves good quality of nursing care at current levels of coverage by increasing nursing staff with a mix of neonatal nurses and NHCAs.	Neonatal nurses and NHCAs	6 in standard care 3 in intermediate care 1 in intensive care	100%	39%
Model 4	This model achieves good quality of nursing care for all newborns in need for public hospital care by increasing nursing staff with a mix of neonatal nurses and NHCAs.	Neonatal nurses and NHCAs	6 in standard care 3 in intermediate care 1 in intensive care	100%	71%
Model 5	This model achieves moderate quality of nursing care at current levels of coverage by increasing nursing staff with a mix of neonatal nurses and NHCAs.	Neonatal nurses and NHCAs	12 in standard care 6in intermediate care 3 in intensive care	50%	39%

All models assume the same health facilities and therefore, the maximum possible coverage in public hospitals is 71%.

NHCAs, neonatal healthcare assistants.

As table 1 showsmodel 1 has the lowest level of care quality, current level of need coverage, and similar to model 2 it does not employ NHCAs. Model 3 and model 5 employ NHCAs together with nurses, maintain current levels of coverage and achieve halfway (model 5) and full optimal (model 3) quality of care. Model 4 employs NHCAs and nurses to achieve optimal quality of care to 71% of all newborns in need in Nairobi (ie, maximum possible coverage in public hospitals).

### Workshops with stakeholders

We used a series of three workshops with stakeholders to explore which tasks it might be acceptable for nurses to share with a low-level, non-professional cadre termed the NHCA. In the first, lasting 1 day, a group (n=12) of senior nurses, nurse trainers and paediatricians considered a broad list of nursing tasks generated from nursing manuals, recently developed neonatal nursing standards and expert opinion to develop an initial list of tasks that might be shared.^22^ In a second workshop lasting 2 days nurse policymakers, regulators, educators and practitioners (n=15) reviewed and refined this list using discussion to reach consensus (online supplementary appendix 1).

10.1136/bmjgh-2019-001817.supp1 Supplementary data

We used a third workshop lasting 1 day to elicit opinions on the degree to which these tasks might safely be delegated from neonatal nurses to NHCAs. This expert panel (n=14) consisted of three health policymakers related to neonatal care as well as three paediatricians, six nurses and two managers working in neonatal care in public hospitals. The panel was provided with the previously identified list of neonatal care nursing tasks^22^ and was requested to state the percentage of work linked to each task that could be delegated to NHCAs without reducing the quality of the service and considering the level of severity of illness of the baby. Three levels of severity were considered from the most acutely ill to the most stable (ie, intensive, intermediate and standard) based on recent local consensus.^23^ It was important to consider patient acuity in this way as delegating work linked to the same task, for example, feeding a baby, may be influenced by the condition of the baby, for example, whether or not they are also receiving oxygen. In this third workshop, the authors guided the panel through questions and discussion on each task that was a candidate for sharing with NHCAs and individual responses were collected using an online survey tool on the proportion of work that could be delegated within each category of illness severity. The variation in the responses between the workshop participants provided us with parameter distributions that were used in the simulation model.

In a separate exercise, participants in the second workshop developed an initial outline of an NHCA training course and suggested a proposed salary level for an NHCA based on existing Government of Kenya public sector recommendations spanning skilled and unskilled workers in hospitals. This proposed NHCA salary was also discussed at the third workshop and a consensus opinion agreed on a suitable value to be included in models reached.

### Simulation model

Monte-Carlo simulation with 20 000 iterations was performed for each model to estimate: (1) the number of newborns treated in public hospitals, (2) the number of nurses and NHCAs, (3) number of effectively treated newborns (the assumptions underlying this are presented below) and (4) neonatal nursing staff costs. Salaries and training costs were adjusted for inflation using the average annual inflation rate between 2020 and 2025 as projected by the International Monetary Fund.^24^ Following guidelines of the WHO,^25^ these were estimated over a period of 10 years (between 2020 and 2029) and discounted to 2017 values using a 3% rate. Neonatal nursing staff costs included the cost of training and supervising NHCAs and were estimated using the government’s perspective. Efficiency is expressed in terms of average cost per newborn effectively treated in public hospitals and number of newborns effectively treated in public hospitals per 100 000 Kenyan Shillings (KES) (US$1000) and is graphically presented as the percentage effectively treated newborns (of all newborns in need for public hospital care) by different levels of neonatal nursing staff budget.

In the simulation model, it was assumed that (1) a task was delegated from nurses to NHCAs only if it can be performed equally well (note that this was part of the instructions in the workshops), (2) the quality of neonatal nursing care was proportional to staff-to-newborn ratio, (3) effectively treated newborn was defined when 80% of the required tasks per care category were performed,^22 23^(4) productivity levels of nurses were constant between 2020 and 2029, (5) the distribution of newborns across the categories of severity of care remained constant between 2020 and 2029, (6) the birth rate in Nairobi remained constant between 2020 and 2029, (7) the share of public hospital admissions remained the same between between 2020 and 2029, (8) the severity of the untreated newborns followed the same distribution as in the treated newborns, when considering increase in coverage, and (9) the nurses freed-up time from delegating tasks to NHCAs was mainly spent to provide neonatal care.

The structure of the model was similar to a decision tree. It first estimated the number of admissions by type of neonatal care and then for each alternative, it estimated sequentially: (1) the number of newborns receiving good quality of care, which was approximated by high staff-to-newborn ratios, (2) the full-time equivalents (FTE) of nurses and NHCAs needed to provide the assumed level of quality of care, (3) the associated nursing staff costs including the costs of NHCAs training, supervision and attrition. These were estimated for each category of care and year.

Population growth rates, the rate of newborns in need for hospital care, the percentage of them receiving hospital care and the proportion of all hospital admissions to public hospitals were included in the model based on estimates from published studies on the Nairobi population.^8 9 19^ Neonatal in-hospital care was categorised by level of severity into standard, intermediate and intensive care in the simulation model. For each category of care, the model input parameters included the number of newborns, admission rate, rate of newborns surviving the first day of admission and length of hospital stay conditional on surviving the first admission day (this was done to account for the high mortality rate on day 1 and to decrease the skewness in hospital days). These parameters as well as the transition of newborns from the most severe to the least severe category of care were informed by data from one of the four public hospitals in Nairobi for which high-quality data exist and confirmed by the experts during the workshops.^26^

The current staff-to-patient ratio and the percentage of newborns receiving currently optimal care (ie, at least 80% of required tasks to have been performed) as well as the desired local aims for staff-to-patient ratio in each category of care to deliver optimal care were based on published estimates.^19 23 27^ The NHCA training costs were agreed by the panel experts during the workshops (based on the government subsidy to Kenya Medical Training college for other cadres but prorated based on the assumption on how long NHCA would be trained) and nursing staff salaries were retrieved from sources of the Government of Kenya. Attrition rates of NHCAs were assumed to be equal to those of nurses reported in the literature.^28^ All input parameters (except for the proportion of task delegation) and their sources are presented in online supplementary appendix 2.

In addition to the probabilistic estimates provided by the simulation model (based on the uncertainty in the input parameters presented in online supplementary appendix 2 and in the proportion of task delegation), we performed two univariate sensitivity analyses. In the first, the rate of newborns needing hospital care over the next 10 years was reduced by 20% to reflect improvements in population health due to public health interventions and decreased socioeconomic deprivation. In the second, we assessed the impact of increased hospital length of stay assuming that optimal neonatal care would reduce in-hospital mortality rates after surviving the first hospital day. This was done by doubling the length of hospital stay in newborns admitted to intensive care as this was the group with the highest mortality rate. These univariate sensitivity analyses were performed using model 2 and model 3.

### Patient and public involvement

No patients were involved in this simulation study.

## Results

### Potential skill-mix change in neonatal nursing care

The results from the workshops with the experts about the tasks that could be safely delegated to NHCAs are presented in table 2. The first two columns provide identified nursing tasks in neonatal care and the proportion of a nurse FTE required to be performed.^22^ As shown in the next three columns, complex tasks (eg, managerial tasks and emergency cases) were assumed to remain entirely in the responsibility of nurses. The expert panel agreed that 85% of nurse tasks in standard care (where babies receive few interventions and are largely recovering from illness or having their basic needs provided for) could be safely delegated to NHCAs. This percentage was perceived to be lower in intermediate care and intensive care.

**Table 2:** Proportion of tasks to be safely delegated from neonatal nurses to NHCAs

Task	% of nurse FTE	% of task delegated to NHCA			% of nurse FTE delegated to NHCA		
		Standard care	Intermediate care	Intensive care	Standard care	Intermediate care	Intensive care
Administrative duties,for example, allocating duties, billing, attending meetings, collecting or ordering supplies and so on	0.051	0.00	0.00	0.00	0.00	0.00	0.00
Admission and discharge of the babies into and out of the neonatal unit	0.060	0.85	0.42(0.08)	0.22(0.04)	0.05	0.02	0.01
Attending continuous medical education (CME) meetings	0.047	0.00	0.00	0.00	0.00	0.00	0.00
Attending ward rounds and doing follow-up of patient care for the babies,for example, booking tests for babies	0.061	0.85	0.02(0.01)	0.03(0.02)	0.05	0.00	0.00
Cleaning and preparing feeding equipment/utensils for the babies	0.046	0.85	0.74(0.07)	0.59(0.09)	0.04	0.03	0.03
Conducting hygiene and infection control activities within the unit,for example, hand washing, proper disposal of waste	0.059	0.85	0.71(0.07)	0.58(0.09)	0.05	0.04	0.03
Counselling mother on KMC and breastfeeding and communicating to mothers on baby's condition/care	0.065	0.85	0.44(0.07)	0.34(0.07)	0.06	0.03	0.02
Dealing with emergencies,for example, resuscitating a baby	0.085	0.00	0.00	0.00	0.00	0.00	0.00
Documenting care and treatment for the babies in the unit	0.077	0.85	0.45(0.09)	0.27(0.07)	0.07	0.03	0.02
Ensuring the babies are comfortable,that is, bathing babies, preparing their linen and making their beds	0.054	0.85	0.74(0.05)	0.33(0.09)	0.05	0.04	0.02
Handover of the babies and equipment during shift changes	0.057	0.85	0.46(0.09)	0.33(0.07)	0.05	0.03	0.02
Monitoring input/output of fluids and feeds for the babies in the unit requiring it	0.061	0.00	0.45(0.10)	0.25(0.08)	0.00	0.03	0.02
Preparing and administering medication and intravenous fluids for the babies in the unit	0.074	0.00	0.00	0.00	0.00	0.00	0.00
Preparing feeds and feeding the babies via cup or NG tube for the babies requiring it	0.057	0.85	0.47(0.09)	0.35(0.08)	0.05	0.03	0.02
Teaching, supervising and mentoring students and other staff	0.069	0.00	0.00	0.00	0.00	0.00	0.00
Vital sings monitoring and regular assessment of condition of the babies in the unit	0.077	0.85	0.29(0.06)	0.13(0.05)	0.07	0.02	0.01
Total	1.000				0.49	0.31	0.20

In brackets are presented the SEs of the mean estimates elicited during the experts workshop; the proportion of a nurse FTE is derived from a survey and validated using expert opinion.

FTE, full-time equivalents; KMC, kangaroo mother care; NG, nasogastric tube; NHCAs, neonatal healthcare assistants.

Overall, it was perceived that 49% of a nurse FTE could be delegated to NHCAs in locally defined forms of standard care, 31% in intermediate care and 20% in intensive care (the technological sophistication of intermediate and intensive dare defined for this setting is much lower that for high-income settings, for further information, see Keene *et al*).^23^

### Effective coverage and costs

As table 3 shows, 122 963 (95%CI78 068 to 179 490) newborns are projected to be admitted to the four public hospitals in Nairobi over 10 years (assuming an annual growth rate of 3%) and 9 (95%CI4 to 15) additional nurses are estimated to be needed to keep current levels of quality of neonatal care (model 1). As very few babies receive quality of care under model 1, there would only be 1060 (95%CI657 to 1596) effectively treated newborns over 10 years at a cost of 455 (95%CI231 to 775) million KES (US$4.5 million) for neonatal nursing staff. Providing quality care and keeping current coverage levels by employing 183 (95%CI113 to 275) additional nurses (model 2) would result to 107 033 (95%CI67 966 to 1 56 207) effectively treated newborns and cost 3104 (95%CI1908 to 4728) million KES (US$31.0 million) over 10 years. Achieving the same quality levels (at current coverage) but with a mix of nurses and NHCAs (model 3), would require to employ 129 (95%CI79 to 195) additional nurses and 74 (95%CI46 to 113) NHCAs. This would result to the same number of effectively treated newborns as in model 2 but at 2558 (95%CI1574 to 3888) million KES (US$25.6 million) over 10 years. According to the estimates of model 4, providing quality care to all newborns in need for (public) hospital care using a new skill-mix, would require 360 (95%CI247 to 506) additional nurses and 189 (95%CI128 to 270) NHCAs over 10 years. This would lead to 191 369 (95%CI1 25 779 to 271 036) effectively treated newborns and 6089 (95%CI4148 to 8614) million KES (US$60.9 million) in nursing staff costs over 10 years. Improving staff-to-patient ratios halfway between current and optimal level using nurses and NHCAs (model 5) would result to 53 522 (95%CI33 986 to 78111) effectively treated newborns and 1117 (95%CI686 to 1691) million KES (US$11.2 million) over 10 years. The parameter uncertainty in the estimated staff costs and effectively treated newborns in each model are graphically presented in online supplementary appendix 3.

**Table 3:** Results of the main simulation analysis

Outcomes and costs	Model 1	Model 2	Model 3	Model 4	Model 5
Total number of newborns treated in public hospitals over 10 years	122 963 (78068 to 179490)	122 963 (78068 to 179490)	122 963 (78068 to 179490)	222 192 (152146 to 306743)	122 963 (78068 to 179490)
Total number of additional nurses to be employed in public hospitals over 10 years	9 (4 to 15)	183 (113 to 275)	129 (79 to 195)	360 (247 to 506)	43 (26 to 68)
Total number of NHCAs to be employed in public hospitals over 10 years	0	0	74 (46 to 113)	189 (128 to 270)	35 (21 to 54)
Total number of newborns effectively treated in public hospitals over 10 years (discounted)	1060 (657 to 1596)	107 033 (67966 to 156207)	107 033 (67966 to 156207)	191 369 (125779 to 271036)	53 522 (33986 to 78111)
Total neonatal nursing staff cost in public hospitals over 10 years (discounted, in million KES)	455 (231 to 775)	3104 (1908 to 4728)	2558 (1574 to 3888)	6089 (4148 to 8614)	1117 (686 to 1691)
Average cost per newborn effectively treated in public hospital (KES)	421 621 (284342 to 614557)	28 744 (23130 to 36078)	23 804 (19058 to 29883)	31 296 (23802 to 43893)	20 815 (16795 to 25783)
No of newborns effectively treated in public hospitals per 100000 KES	0.24 (0.16 to 0.35)	3.48 (2.77 to 4.32)	4.20 (3.35 to 5.25)	3.20 (2.28 to 4.20)	4.80 (3.88 to 5.95)

95% CIs are presented in brackets; time horizon: 2020–2019; the estimated results are just for Nairobi County (population of 4.5 million in 2019) and the public sector (assuming it continues to provide 71% or care across sectors).

KES, Kenyan Shillings.

Compared with the estimates of model 1 and model 2, the required nurses and NHCAs to deliver optimal care as well as the associated staff costs decreased by 20% when less need for hospitalisation was assumed (ie, first sensitivity analysis) and increased by ~35%, respectively, when a length of hospital stay was assumed to be double in newborns admitted to intensive care due to improved survival rates (ie, second sensitivity analysis). These results are presented in table 4.

**Table 4:** Results of the univariate sensitivity analyses

Outcomes and costs	Reduced need for hospitalisation		Increased length of stay in intensive care	
	Model 2	Model 3	Model 2	Model 3
Total number of newborns treated in public hospitals over 10 years	98 511 (62683 to 143629)	123 295 (78870 to 179247)	122 963 (78068 to 179490)	122 963 (78068 to 179490)
Total number of additional nurses to be employed in public hospitals over 10 years	146 (90 to 221)	129 (80 to 194)	250 (154 to 380)	190 (115 to 291)
Total number of NHCAs to be employed in public hospitals over 10 years	0	75 (46 to 113)	0	87 (54 to 131)
Total number of newborns effectively treated in public hospitals over 10 years (discounted)	85 749 (54588 to 124952)	85 749 (54588 to 124952)	107 033 (67966 to 156207)	107 033 (67966 to 156207)
Total neonatal nursing staff cost in public hospitals over 10 years (discounted, in million KES)	2487 (1522 to 3771)	2049 (1258 to 3103)	4124 (2511 to 6292)	3534 (2147 to 5407)
Average cost per newborn effectively treated in public hospital (KES)	28 744 (23131 to 36170)	23 804 (15236 to 23708)	38 174 (30137 to 49188)	32 762 (25554 to 42657)
No of newborns effectively treated in public hospitals per 100000 KES	3.48 (2.76to4.32)	4.20 (3.36to5.24)	2.62 (2.03to3.32)	3.05 (2.34to3.91)

KES, Kenyan Shillings.

**Figure 1:**
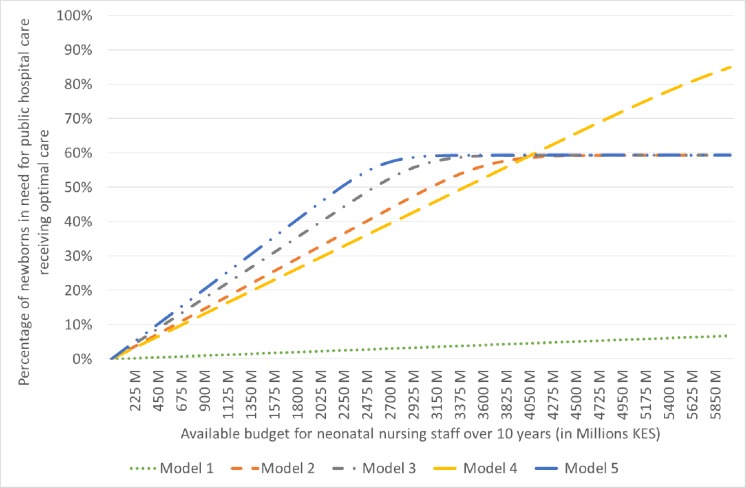
Effective coverage of newborns by level of budget (KES) for neonatal nursing staff.The curves of models 2, 3 and 5 flatten at some point because these models assume current levels of coverageirrespective of budget level. KES, Kenyan Shillings.

Comparing the different models based on efficiency at current coverage levels, model 5 appears to lead to the lowest mean cost per newborn effectively treated in public hospitals (mean: KES 20815 (US$280); 95% CI16 795 to 25783) and the highest number of newborns effectively treated in public hospitals per 100000 KES (US$1000) (mean: 4.80; 95% CI3.88 to 5.95). However, this model fails to address the current coverage gap. Figure 1 presents the effective coverage of all newborns in need for hospitalisation in public hospitals at different levels of nursing staff budget. As the figure shows, model 5 would require ~2.9 billion KES (US$29 million) to provide quality care to 59% of all newborns in need for public hospital care (ie, current level of coverage) over the period of 10 years followed by model 3 that would require about 3.1 billion KES (US$31 million) for the same result. If there was more budget available for nursing staff over 10 years, then model 4 would require ~6.6 billion KES (US$66 million) to provide quality of care to >90% of all newborns in need for hospital care. In other words, figure 1 shows that it is more efficient to provide modest quality of care to more newborns by using a mix of nurses and NHCAs (model 5) if the available budget for neonatal nursing staff costs over 10 years is around 3 billion KES (US$30 million). Above that budget level, the authorities should consider expanding the levels of neonatal care coverage while keeping the quality of care at optimal levels.

## Discussion

This exploratory study provides the first impact estimates of introducing new nurses and NHCAs into public hospitals in Nairobi City County on effective coverage (resulting from an assumed direct relationship between staff ratios and quality) and neonatal nurse staff budgets. It appears that a combination of nurses and NHCAs is likely to be the most affordable option to improve the quality of neonatal care within any given budget for nursing staff costs.

Even if NHCAs were to be introduced in neonatal care as part of an effort to improve quality without changing coverage there will still be a requirement to almost double the number of nurses employed to ensure high-quality of care. This is because the main driver for improving quality in neonatal care in our models is improving nurse to baby ratios in settings with severe workforce deficits at baseline; a proposition supported by recent observational research.^27^ Yet, it should be noted that even the most cost-effective strategies with a mix of new nurses and NHCAs would still result in staff having to care for far more babies than is currently acceptable in many high-income settings (although the range of interventions provided in these LMIC settings is also more limited).^23^

These results have two important implications. First, while introducing task-shifting appears cost-effective this depends on NHCAs being able to carry out certain tasks as well as nurses and their scope of work. Both of these issues would benefit from further study. Second, if workforce deficits are as we believe a key factor undermining the delivery of quality care then in Nairobi City County in Kenya, which at ~4.5 million people represents about 10% of the Kenyan population, a minimum investment of US$26 million (<US$0.6 per person per year in population terms) over the next decade will be required in nurse staffing to improve quality without addressing major existing coverage gaps. To address both coverage and quality gaps would require a US$61 million investment over 10 years in the nursing workforce and likely additional investment in infrastructure and other forms of staffing. Such investments are, we believe, likely to be needed to dramatically improve newborn survival in high mortality settings.

It is acknowledged that the introduction of healthcare assistants in healthcare is challenging and may create tension with nurses about occupational interests.^29 30^ However, healthcare assistants are well embedded in the healthcare systems of high-income countries and their importance is expected to grow.^31^Healthcare assistants are also increasingly used in private hospitals in LMICs.^32^ Despite this trend, the evidence about the effectiveness and cost-effectiveness of healthcare assistants is scarce.^33^ A recent economic evaluation concluded that delegating tasks from medical doctors to physician assistants in 34 hospital wards across the Netherlands may reduce staff costs without jeopardising quality care.^34^ In a literature review of 48 economic evaluations of strategies to improve maternal care in LMICs, none of the studies assessed healthcare assistants in the community or hospital setting (although community health workers were included).^35^ This highlights the need for evidence about the effectiveness and cost-effectiveness of NHCAs to support policymakers in considering the introduction of NHCAs as an option to reduce neonatal mortality.

This analysis is focusing on one aspect of a complex and multifaceted problem on how to improve neonatal care in LMICs. The assessed alternative strategies were limited to nurses and NHCAs without considering possible task delegation to mothers or other staff cadres. While task-shifting to mothers may appear an attractive, low-cost option it should be recognised that to achieve this, for a population of sick newborns at high risk of death, staff would need to spend considerable amounts of time educating, supervising and supporting mothers to take up such roles. The work we have conducted on delivery of existing care (model 1), where nurses are responsible for 15 or more babies, suggests they do not have time to support mothers to give safe, effective care.^27 36^ Efforts to promote family centred care for the sickest of babies should not therefore be confused with the desperate shifting of tasks to mothers that results from critical workforce shortages.

Policymakers in LMICs may also want to consider other health services interventions to improve quality of neonatal care. However, a recent Cochrane review of systematic reviews on delivery arrangements for health systems in LMICs found that many interventions are focusing on new roles and task shifting.^37^ These included community-based neonatal packages with additional training of outreach workers, lay health workers to deliver care for mothers and children, non-physician providers for abortion care, health workers providing social support during at-risk pregnancies, and midwife-led care for childbearing women and their infants. However, virtually none of these task-shifting interventions were in hospital settings and the assessment of an even broader set of skill-mix configurations in neonatal hospital care was beyond the scope of this study.

The explorative nature of our study has similar characteristics with early health technology assessment (HTA) that provides economic evidence to drug and device manufacturers during early stages of clinical research (eg, to provisionally test the potential cost-effectiveness of a promising idea or molecule before starting investment in researchanddevelopment).^38^ Early HTA is, however, particularly challenging in the evaluation of complex interventions^39^ as well as in in LMICs for several reasons including technical capacity and data.^40^ We also faced these challenges in our study and tried to employ the best possible research methods to overcome them (such as organising workshops with experts, getting access to hospital records when available, and using mainly recently published literature from the same setting as this simulation study is part of a broader study). The need for HTA to inform rational priority setting in LMICs, where it is virtually non-existent, is urgent.^41^ Hopefully more explorative economic studies will be conducted in these settings to inform the allocation process of scarce resources at an early stage.

The main strengths of this study are the use of published data in combination with primary data derived from workshops with experts for most input parameters in the simulation model as well as the probabilistic and univariate analyses that address the parameter uncertainty in the results.

The study has also several limitations with first and foremost the quality of the data used as the input parameters in the simulation model. Due to lack of data, we assumed a linear relationship between patient–staff ratios and quality of neonatal care without considering possible diminishing return on investment. However, this is less problematic when considering the relative performance of each skill-mix configuration in our study (ie, comparing the estimated effectively covered newborns and associated costs between the different models). In addition, a linear relationship might still hold considering that any diminishing return would be realised far beyond the current staff-to-newborn ratio, which was very low.

Another limitation is that the study ignores any needed increase in physical capacity and resources (eg, consumables) of facilities and the costs of administration of a larger workforce. The four public hospitals included in this study already have very high bed occupancy so, new or upgraded facilities along with the redesign of antenatal, delivery and neonatal care services would also likely be needed.^23^ The estimates of our study though provide the required budget for staff costs if authorities considered a strategic plan to enable effective coverage.

Last, there are many factors that may influence neonatal nursing staff workload, influencing therefore quality of care, that are not incorporated in the simulation model. Such factors include birth rate, changing environment (eg, sanitation) and change in socioeconomic status. It is even unknown whether improvements in quality of care would increase hospital length of stay because of improved early survival (especially of preterm babies who tend to have very long stays) or reduce length of stay because of more rapid resolution of illness. Although the two univariate analyses attempted to incorporate some of these factors in the analysis, the reality is be much more complex and unpredictable.

## Conclusion

Changing skill-mix in hospital care by introducing NHCAs to work with additional nurses may be an affordable way to improve quality of hospital care for newborns and so reduce neonatal mortality in LMICs. This option should be considered in ongoing policy discussions and supported by further evidence. The relevance of our work in other areas of inpatient care provision should also be explored.
