# Supplementary material for: Effective coverage and budget implications of skill-mix change to improve neonatal nursing care: an explorative simulation study in Kenya
Source: BMJ Glob Health. 2019 Dec 2;4(6):e001817. doi: 10.1136/bmjgh-2019-001817 (PMC6936475; doi:10.1136/bmjgh-2019-001817)

Appendix 1 Potential areas a NHCA might support nurses to undertake tasks

Area	Tasks
1. Infection prevention and control	a. Cot cleaning / disinfection<br>b. Incubator cleaning / disinfection<br>c. Promoting hand hygiene and other infection prevention and control measures amongst mothers and family members (visitor's education)<br>d. Cleaning / sterilising cups and other basic utensils<br>e. Waste segregation/disposal<br>f. Use of protective gears
2. Routine baby care	a. Cord care (chlorhexidine)<br>b. Initiating and supporting mothers as part of Kangaroo Mother Care (KMC):<br>i. Appropriate positioning / baby & mother comfort<br>ii. Supporting mother's continued feeding (Nasal Gastric Tube (NGT), cup and spoon, breast feeding)<br>c. Basic education and emotional support<br>i. Mother's self-care (breast pain / redness; personal and family hygiene, nutrition etc)<br>ii. Baby care – helping family to learn to care for baby (bathing, clothing, hygiene and care of nappy area and skin, changing diapers, cord-care, etc)<br>iii. Empathetic care for mothers / families in distress<br>d. Health promotion (as part of team-based approach led by nurses):<br>i. Reinforcement of messages on Anti-retroviral (ARV) therapy adherence where relevant<br>ii. Reinforcement of HIV and Sexually transmitted Infection (STI) prevention<br>iii. Supporting education on family planning<br>iv. iv.Reinforcing nurse led discharge instructions
3. Contribution to feeding	a. If clinicians / nurses have initiated NGT or cup and spoon feeding to support:<br>i. Helping the mother express to provide the recommended amount of expressed breast milk (EBM)<br>ii. Preparing formula according to standard guidance<br>iii. Measure EBM / formula prior to each ordered feed so feeds accurately given to each baby<br>iv. Labelling and storing of milk in fridge / freezer ensuring baby receives mother's milk or correct formula<br>b. Education / support role to mothers for feeding:<br>i. Instruction on expressing breast milk / importance of breast milk<br>ii. Instruction on routine breast care / hygiene (e.g. to prevent cracked nipples, engorgement)<br>iii. Instruction on maternal self-care on own nutrition / fluid intake
4. Contribution to general ward duties	a. Changing bed-linens<br>b. Support to mother / radiographer to position baby for simple X-ray being conducted on ward<br>c. Completing checklist of basic equipment in ward section to support effective handover<br>d. Recording of stocks of non-pharmaceuticals at end of day / shift as directed by nurse in charge<br>e. Accompany stable baby for routine visits to X-ray / Lab (e.g. long-stay baby requiring no supportive care for repeat chest X-ray (at discretion of nurse in charge)<br>f. Collecting supplies / medicines from stores / pharmacy etc as directed by nurse in charge
5. Contribution to basic monitoring and assessment	a. Together with regular activities such as baby feeding / bathing to be capable of checking for possible signs of illness so these can be reported to the nurse in charge:<br>iv. Specific skin signs (e.g. rashes, ulcers, bruising)<br>v. Jaundice<br>vi. Possible indicators of severe illness (e.g. increasing breathing difficulty)<br>vii. Signs of problems at indwelling cannula sites (e.g. redness, swelling)

		b. Weighing babies and recording weight on monitoring chart
6.	Promoting safe and effective delivery of basic interventions	a. If clinicians / nurses have initiated phototherapy to support:<br>i. Applying eye shields<br>ii. Checking eye shielding is safely applied and where opportunities (e.g. during feeding) check eyes for redness<br>iii. Intermittent monitoring (for example after replacing under lights post-feeding or at times of monitoring vital signs) to ensure adequate exposure to lights and positioning changed<br>iv. Indicating on monitoring chart that phototherapy being given<br>b. For babies on oxygen:<br>i. Checking that nasal prongs or nasal catheter correctly positioned<br>ii. Checking nostrils are not blocked by mucus<br>iii. Checking no damage to skin from nasal prongs / catheter<br>c. For babies on oral fluids / feeds:<br>i. Helping nurses with basic measures of output (e.g. wet nappies, stool passed)<br>d. Input to ward round on general progress of babies for whom they are providing supported care as requested by clinical or nursing team
7.	Roles as a first responder in case of emergency	a. Awareness of indications of possible serious illness and need to seek urgent help from senior staff<br>b. Ability to act as ‘first responder’ in cases where senior help is not immediately available, examples:<br>i. Positioning baby if convulsing to avoid aspiration<br>ii. In cases where a baby collapses and expert help is not immediately at hand should be able to position airway and be provide effective BVM for critical few minutes until expert help arrives<br>iii. Sufficient understanding of basic emergency equipment enabling them to identify and collect items for professional staff managing an emergency<br>c. Emotional support / care for family if present during emergency care

Appendix 2 Input parameters used in the simulation model

	Mean	Standard error	Distribution	Alpha	Beta	Source
**Population**						
Nairobi city crude birth rate	0.031	0.003	Beta	96	3029	Murphy et al., 2018[8]
Annual population growth	0.039	0.004	Beta	95	2375	Murphy et al., 2018[8]
Nairobi population (2017)	4260000					Murphy et al., 2018[8]
**Hospitalisation**						
% of newborns in need for hospital care	0.18	0.02	Beta	81	364	Murphy et al., 2018[8]
% of newborns admitted to public hospitals	0.71	0.07	Beta	28	12	Murphy et al., 2018[9]
% of admissions to standard care	0.74	0.07	Beta	25	9	Hospital records[25]
% of admissions to intermediate care	0.13	0.01	Beta	86	582	Hospital records[25]
% of admissions to intensive care	0.13	0.01	Beta	86	582	Hospital records[25]
Mean days of stay in intensive care after admitted to intensive care	3.00	0.30	Gamma	100	0	Expert opinion based on hospital records[25]
Mean days of stay in intermediate care after admitted to intensive care	2.00	0.20	Gamma	100	0	Expert opinion based on hospital records[25]
Mean days of stay in standard care after admitted to intensive care	1.00	0.10	Gamma	100	0	Expert opinion based on hospital records[25]
Mean days of stay in intermediate care after admitted to intermediate care	3.00	0.30	Gamma	100	0	Expert opinion based on hospital records[25]
Mean days of stay in standard care after admitted to intermediate care	2.00	0.20	Gamma	100	0	Expert opinion based on hospital records[25]
Mean days of stay in standard care after admitted to standard care	3.50	0.35	Gamma	100	0	Expert opinion based on hospital records[25]
**In-hospital mortality**						
Probability of 1-day survival after admission to intensive care	0.860	0.020	Beta	258	42	Hospital records[25]
Probability of 1-day survival after admission to intermediate care	0.960	0.006	Beta	1023	43	Hospital records[25]
Probability of 1-day survival after admission to standard care	0.990	0.001	Beta	15313	155	Hospital records[25]
**Current care (Model 1)**						
Average number of newborns per nurse in public hospitals	15.24	1.52	Normal			Murphy et al., 2018[19]
% untreated births in need for care	0.45	0.04	Beta	55	69	Murphy et al., 2018[9]
% of low need newborns receiving good quality nursing care	0.01	0.00	Beta	98	9801	Gathara et al., 2019[26]
% of medium need newborns receiving good quality nursing care	0.01	0.00	Beta	98	9801	Gathara et al., 2019[26]
% of high need newborns receiving good quality nursing care	0.01	0.00	Beta	98	9801	Gathara et al., 2019[26]
**Optimal care for current proportion of treated babies (Models 2 & 3)**						
Mean number of babies per nurse (low need)	6.00	0.60	Normal			Keene et al., 2019[23]
Mean number of babies per nurse (medium need)	3.00	0.30	Normal			Keene et al., 2019[23]
Mean number of babies per nurse (high need)	1.00	0.10	Normal			Keene et al., 2019[23]
% of low need newborns receiving good quality nursing care	1.00					Workhops
% of medium need newborns receiving good quality nursing care	1.00					Workhops
% of high need newborns receiving good quality nursing care	1.00					Workhops
**Optimal care for all babies in need for inpatient care (Model 4)**						
% untreated births in need for care	0.00					Workhops

**Semi-optimal care for current proportion of treated babies (Model 5)**						
Mean number of babies per nurse (low need)	12	1.20	Normal			Scenario: half-way improvement
Mean number of babies per nurse (medium need)	6	0.60	Normal			Scenario: half-way improvement
Mean number of babies per nurse (high need)	3	0.30	Normal			Scenario: half-way improvement
% of low need newborns receiving good quality nursing care	0.50					Scenario: half-way improvement
% of medium need newborns receiving good quality nursing care	0.50					Scenario: half-way improvement
% of high need newborns receiving good quality nursing care	0.50					Scenario: half-way improvement
**Salaries, training, and supervision**						
Annual gross salary of enrolled community nurse (KES)	1182720	118272	Normal			Government of Kenya, confirmed in workshops
Annual gross salary of nurse with basic diploma (KES)	1466520	146652	Normal			Government of Kenya, confirmed in workshops
Annual gross salary of nurse with higher diploma (KES)	2341320	234132	Normal			Government of Kenya, confirmed in workshops
Annual gross salary of nurse with degree (KES)	1886400	188640	Normal			Government of Kenya, confirmed in workshops
Annual gross salary of degree intern nurse (KES)	1073568	107357	Normal			Government of Kenya, confirmed in workshops
Proportion of enrolled community nurses	0.11					Murphy et al., 2018[19]
Proportion of nurses with basic diploma	0.59					Murphy et al., 2018[19]
Proportion of nurse with higher diploma	0.18					Murphy et al., 2018[19]
Proportion of nurses with degree	0.11					Murphy et al., 2018[19]
Proportion of degree intern nurses	0.01					Murphy et al., 2018[19]
Annual gross salary of health care assistant, years 0-3 (KES)	30000	3000	Normal			Workshops
Annual gross salary of health care assistant, years 4-6 (KES)	34490	3449	Normal			Workshops
Annual gross salary of health care assistant, years 7-10 (KES)	35620	3562	Normal			Workshops
Health care assistant training cost (KES per person)	10000	1000	Normal			Workshops
% of nurse time to be dedicated to NHCA supervision	0.1	0.01	Beta	89	810	Workshops
Attrition rate of nurses and NHCAs	0.072	0.01	Beta	92	1196	Lopes et al., 2017[27]; assumed to be equal to nurses
Inflation rate	0.050	0.001	Normal			International Monetary Fund- average projection 2020-2024
**Other parameters**						
Discount rate for costs and outcomes	0.03					WHO guidelines[24]
Standard error as percentage of the mean	0.1					Assumption
Increase rate in length of hospital stay due to higher in-hospital survival	2					Assumption
Staff shifts per day	3					Murphy et al., 2018[19]
Annual working days in a full-time equivalent	220					Expert opinion

Note: Nurse salaries include all allowances and benefits

Appendix 3 Uncertainty around the average cost-effectiveness of the alternatives

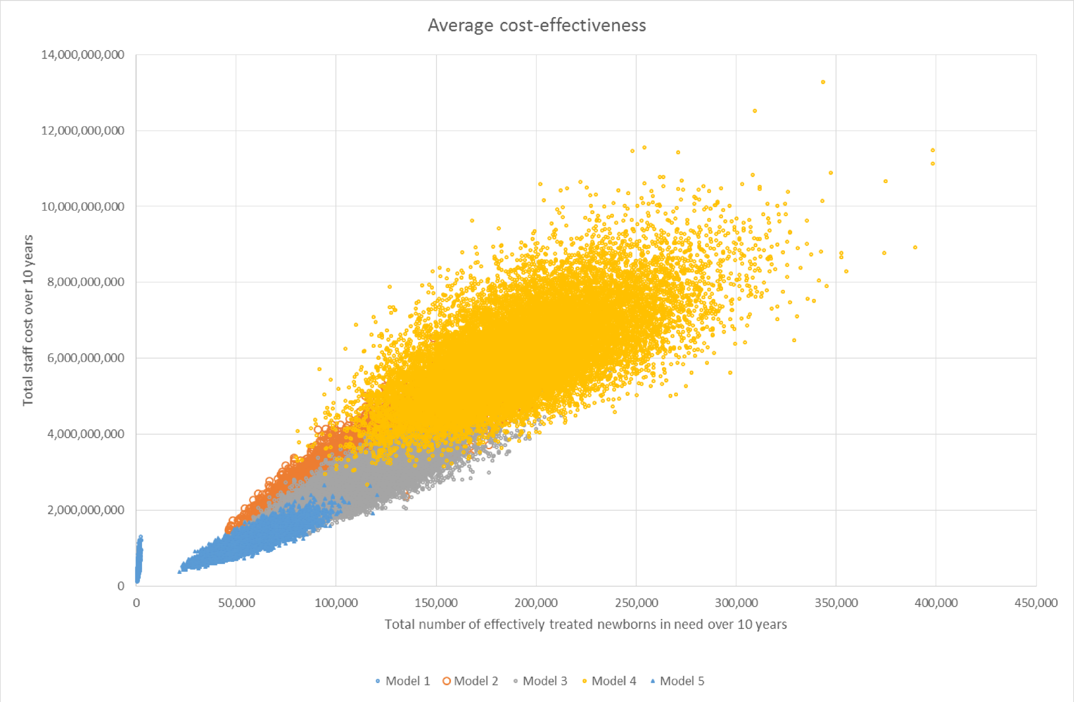

Supplement: Supplementary data [file bmjgh-2019-001817supp001.pdf]
